# Supplementary material for: Anti-Inflammatory Properties of Eugenol in Lipopolysaccharide-Induced Macrophages and Its Role in Preventing β-Cell Dedifferentiation and Loss Induced by High Glucose-High Lipid Conditions
Source: Molecules. 2023 Nov 16;28(22):7619. doi: 10.3390/molecules28227619 (PMC10673503; doi:10.3390/molecules28227619)
Supplement: Supplementary file 1 [file molecules-28-07619-s001.zip › molecules-2709775-supplementary.pdf]

Supplementary materials

Table S1. The information of primary antibodies used in the experiments

Primary antibody	Manufacture	Cat #	Dilution
Nlrp3	Cell Signaling Technology	15101S	1:1000
Phospho-NFκB-p65 (ser536)	Cell Signaling Technology	13346S	1:1000
NFκB p-65	Santa Cruz Biotechnology	sc-8008	1:200
Phospho-TYK 2 (Tyr-1054/1055)	Cell Signaling Technology	9321S	1:500
TNF-α	Cell Signaling Technology	3707	1:500
PANX-1	Cell Signaling Technology	91137S	1:1000
Cox-2	Cell Signaling Technology	12282	1:1000
β-actin	Santa Cruz Biotechnology	sc-47778	1:200
GAPDH	Santa Cruz Biotechnology	sc-32233	1:200
Phospho-STAT3(Tyr-705)	Santa Cruz Biotechnology	sc-8059	1:200
STAT3	Santa Cruz Biotechnology	sc-8019	1:200
Pro-Caspase1	Santa Cruz Biotechnology	sc-392736	1:200
IL-1β	Abcam	ab216995	1:500
β-Actin	Abcam	ab8227	1:2000
GAPDH	Cell Signaling Technology	2118	1:1000
α-Tubulin	Santa Cruz Biotechnology	sc-8035	1:200
IL-6	Cell Signaling Technology	12153	1:1000
TYK2	Cell Signaling Technology	14193S	1:1000
Caspase-3	Cell Signaling Technology	9662s	1:200
Caspase-7	Cell Signaling Technology	12827	1:1000
Cleaved PARP	Cell Signaling Technology	9545s	1:250
PDX-1	Cell Signaling Technology	D59H3	1:1000
FOXO1	Cell Signaling Technology	2880S	1:1000
Phospho-FOXO1 (Ser256)	Cell Signaling Technology	9461S	1:1000
TXNIP	Cell Signaling Technology	14716S	1:1000

Table S2. The sequences of primers used for qRT-PCR.

Rat-Ins1	Forward	GGGAACGTGGTTTCTTCTACA
Rat-Ins1	Reverse	CAGTGCCAAGGTCTGAAGAT
Rat-NEUROD1	Forward	GAACACGAGGCAGACAAGAA
Rat-NEUROD1	Reverse	TCATCTTCATCCTCCTCCTCTC
Rat-MafA	Forward	GGTCATCCGACTGAAACAGAA
Rat-MafA	Reverse	CTTCTCGCTCTCCAGAATGTG
Rat-Ins2	Forward	GGGAGCGTGGATTCTTCTACA
Rat-Ins2	Reverse	AGTGCCAAGGTCTGAAGGT
Rat-Slc2A2	Forward	CATAGTCACACCAGCACATACG
Rat-Slc2A2	Reverse	ACAGACAGAGACCAGAGCATAG
Rat-PDX1	Forward	CCCTTTCCCGTGGATGAAATC
Rat-PDX1	Reverse	GCTGTACGGGTCCTCTTATTCT
Rat-FOXO1	Forward	TCTACGAGTGGATGGTGAAGAG
Rat-FOXO1	Reverse	GGACAGATTGTGGCGAATTGA
Rat-Tubulin	Forward	TGACCCTCGCCATGGTAAATA
Rat-Tubulin	Reverse	GATGGTACGCTTGGTCTTGATG
Human-IL-6	Forward	GGAGACTTGCCTGGTGAAA
Human-IL-6	Reverse	CTGGCTTGTTCCTCACTACTC
Human-COX-2	Forward	TACTGGAAGCCAAGCACTTT
Human-COX-2	Reverse	GGACAGCCCTTCACGTTATT
Human-TNF-α	Forward	CCAGGGACCTCTCTCTAATCA
Human-TNF-α	Reverse	TCAGCTTGAGGGTTTGCTAC
Human-Pannexin-1(PANX-1)	Forward	GTGTGCAGCATCAAATCAGG
Human-Pannexin-1(PANX-1)	Reverse	GACACTGAGCAACTGGAAGA
Human-ADAR1	Forward	GTCATCAATGGCCGAGAGTT
Human-ADAR1	Reverse	CTTGGCTTTGGCTTCCTCTA
Human-Nlrp3	Forward	GAAGAGGAGTGGATGGGTTTAC
Human-Nlrp3	Reverse	TCTGCTTCTCACGTACTTTCTG
Human-IL-1β	Forward	CCTTAGGGTAGTGCTAAGAGGA
Human-IL-1β	Reverse	AAGTGAGTAGGAGAGGTGAGAG
Human-ACTIN	Forward	GGCATCCTCACCCTGAAGTA
Human-ACTIN	Reverse	CACACGCAGCTCATTGTAGAAG
Human-GAPDH	Forward	CAGGAGGCATTGCTGATGAT
Human-GAPDH	Reverse	GAAGGCTGGGGCTCATTT
